# Gemtuzumab Ozogamicin in Acute Myeloid Leukemia: Efficacy, Toxicity, and Resistance Mechanisms—A Systematic Review

**DOI:** 10.3390/biomedicines12010208

**Published:** 2024-01-17

**Authors:** Aurelia Collados-Ros, Manuel Muro, Isabel Legaz

**Affiliations:** 1 Department of Legal and Forensic Medicine, Faculty of Medicine, Biomedical Research Institute (IMIB), Regional Campus of International Excellence “Campus Mare Nostrum”, University of Murcia, 30100 Murcia, Spain; aurelia.c.r@um.es; 2 Immunology Service, University Clinical Hospital Virgen de la Arrixaca, Biomedical Research Institute of Murcia (IMIB), 30120 Murcia, Spain; manuel.muro@carm.es

**Keywords:** acute myeloid leukemia, CD33, gemtuzumab ozogamicin, toxicity, epigenetic therapeutics

## Abstract

Acute myeloid leukemia (AML) is a diverse group of leukemias characterized by the uncontrolled proliferation of clonal neoplastic hematopoietic precursor cells with chromosomal rearrangements and multiple gene mutations and the impairment of normal hematopoiesis. Current efforts to improve AML outcomes have focused on developing targeted therapies that may allow for improved antileukemic effects while reducing toxicity significantly. Gemtuzumab ozogamicin (GO) is one of the most thoroughly studied molecularly targeted therapies in adults. GO is a monoclonal antibody against CD33 IgG4 linked to the cytotoxic drug calicheamicin DMH. The use of GO as a chemotherapeutic agent is not generalized for all patients who suffer from AML, particularly for those whose health prevents them from using intensive conventional chemotherapy, in which case it can be used on its own, and those who have suffered a first relapse, where its combination with other chemotherapeutic agents is possible. This systematic review aimed to comprehensively evaluate GO, focusing on its molecular structure, mode of action, pharmacokinetics, recommended dosage, resistance mechanisms, and associated toxicities to provide valuable information on the potential benefits and risks associated with its clinical use. A systematic review of eight scientific articles from 2018 to 2023 was conducted using PRISMA analysis. The results showed that GO treatment activates proapoptotic pathways and induces double-strand breaks, initiating DNA repair mechanisms. Cells defective in DNA repair pathways are susceptible to GO cytotoxicity. GO has recommended doses for newly diagnosed CD33+ AML in combination or as a single agent. Depending on the treatment regimen and patient status, GO doses vary for induction, consolidation, and continuation cycles. Multidrug resistance (MDR) involving P-glycoprotein (P-gp) is associated with GO resistance. The overexpression of P-gp reduces GO cytotoxicity; inhibitors of P-gp can restore sensitivity. Mitochondrial pathway activation and survival signaling pathways are linked to GO resistance. Other resistance mechanisms include altered pharmacokinetics, reduced binding ability, and anti-apoptotic mechanisms. GO has limited extramedullary toxicity compared to other AML treatments and may cause hepatic veno-occlusive disease (HVOD). The incidence of hepatic HVOD after GO therapy is higher in patients with high tumor burden. Hematological side effects and hepatotoxicity are prominent, with thrombocytopenia and neutropenia observed. In conclusion, GO’s reintroduction in 2017 followed a thorough FDA review considering its altered dose, dosing schedule, and target population. The drug’s mechanism involves CD33 targeting and calicheamicin-induced DNA damage, leading to apoptosis and resistance mechanisms, including MDR and survival signaling, which impact treatment outcomes. Despite limited extramedullary toxicity, GO is associated with hematological side effects and hepatotoxicity.

## 1. Introduction

Cancer is the name given to a set of related diseases. In all types of cancer, some cells in the body begin to divide without stopping and spread to surrounding tissues. It is a significant health problem in our society [1]. Cancers that start in the tissues that make up the blood in the bone marrow are called leukemias [1]. Leukemia is considered acute when cells that have not fully developed are affected, and it is fast evolving. It is myeloid because the cells that undergo the cancer mutation are precursors of blood cells (red blood cells, platelets, and some white blood cells) but not of lymphocytes [2].

It is currently estimated that in 2018, there will be approximately 60,300 new cases of acute myeloid leukemia (AML), of which only 59.6% will survive [3]. However, 50% of affected patients who survive suffer relapses due to resistance generated against chemotherapy [4]. The chemotactic drug gemtuzumab ozogamicin (GO), an anti-CD33 monoclonal antibody coupled with a hydrazide derivative of calicheamicin, the enediyne antibiotic, has produced some of the most promising results to date. Research has focused on the CD33 antigen as a therapeutic target because most AML tumor cells express this marker on their surface, whereas normal hematopoietic stem cells do not [5].

However, the use of GO is not generalized for all patients who suffer AML, particularly for those whose health prevents them from using intensive conventional chemotherapy, in which case it can be used on its own, and for those who have suffered a first relapse, where its combination with other chemotherapeutic agents is possible [3]. This interesting drug obtained accelerated approval at a dose of 9 mg/m^2^ by the Food and Drug Administration [6], and Wyeth Pharmaceuticals have claimed to conduct further clinical trials proving the drug’s benefits. However, it was observed that the response and survival did not increase, and there was a more significant number of deaths with its application, so it was withdrawn from the market in 2010 [7].

After several studies to find out the reason for its reported toxicity, they found that, on the date of approval, the dose was too high and produced hepatic veno-occlusive disease (HVOD), while upon applying doses of 3 to 6 mg/m^2^, the toxicity was considerably lower. Based on these tests, the FDA allowed its use again on 1 September 2017 [8]. The mechanism of action of GO is that it is an antibody against CD33+ cells, so after forming the immunocomplex, it is internalized. After entering the lysosome, the calicheamicin derivative is liberated from the antibody by acid hydrolysis, and by reducing it with glutathione, it forms a reactive intermediate. This intermediate is the one that enters the nucleus and causes explicitly double-stranded breaks in the minor groove of the DNA [9].

During the application of GO as a chemotherapeutic agent, resistance phenomena have been observed against it. What could cause this process are proteins that are expressed in the cell itself and are capable of pumping antileukemic agents into the extracellular medium that enters the malignant cell.

In the case of AML, one of the proteins that produce this resistance is membrane-glycoprotein P (P-gp), specifically the MRP and MDR-1 subfamilies (multidrug resistance proteins). Additionally, because of the drug’s consumption in the peripheral blood and its poor ability to enter the bone marrow tissues, high levels of CD33 tumor burden in the peripheral blood also confer resistance to the treatment and are linked to worse results.

This systematic review aimed to comprehensively evaluate GO, focusing on its molecular structure, mode of action, pharmacokinetics, recommended dosage, resistance mechanisms, and associated toxicities to provide valuable information on the potential benefits and risks associated with its clinical use.

## 2. Systematic Review

The methods used for this systematic review (covering March 2018 to December 2023 were developed with reference to the Preferred Reporting Items for Systematic Reviews and Meta-Analyses (PRISMA) statement [10] for studies published following the methods detailed in the Cochrane Handbook for Systematic Reviews of Interventions [11]. The protocol for this systematic review was registered (ID 495161) with the International Prospective Register of Systematic Reviews (PROSPERO) before commencement. The PRISMA checklist is included in Supplementary Table S1.

### 2.1. Inclusion Criteria

GO, Mylotarg, GO Resistance, CD33, Gemtuzumab Toxicity, Gemtuzumab Leukemia, and Acute Myeloid Leukemia were the keywords and subject headings investigated in all the investigations. Three primary inclusion criteria were used to select the articles: (i) studies on global oncology, (ii) gemtuzumab resistance and toxicity, and (iii) CD33 and acute myeloid leukemia.

### 2.2. Search Strategy

Literature search strategies were developed by a health sciences librarian using two scientific electronic databases (PubMed and Google Scholar) and two grey literature databases (FDA and WHO) and keywords. For the articles included in the review, the following critical characteristics of the studies were identified: topic discussed, first author, and year. The search in the four electronic databases was limited to articles published in English and Spanish. Regarding the inclusion criteria, two impartial reviewers revised full-text publications, abstracts, and titles. The percentage of favorable agreement was used to compute the two reviewers’ interrater agreement for study selection [12].

### 2.3. Data Extraction

Two impartial testers used Microsoft Excel 365 to extract duplicate data. We examined and contrasted several reports from the same investigation, extracting the relevant information where it was available for every study that matched the inclusion requirements.

### 2.4. Descriptive Studies

**Figure 1:**
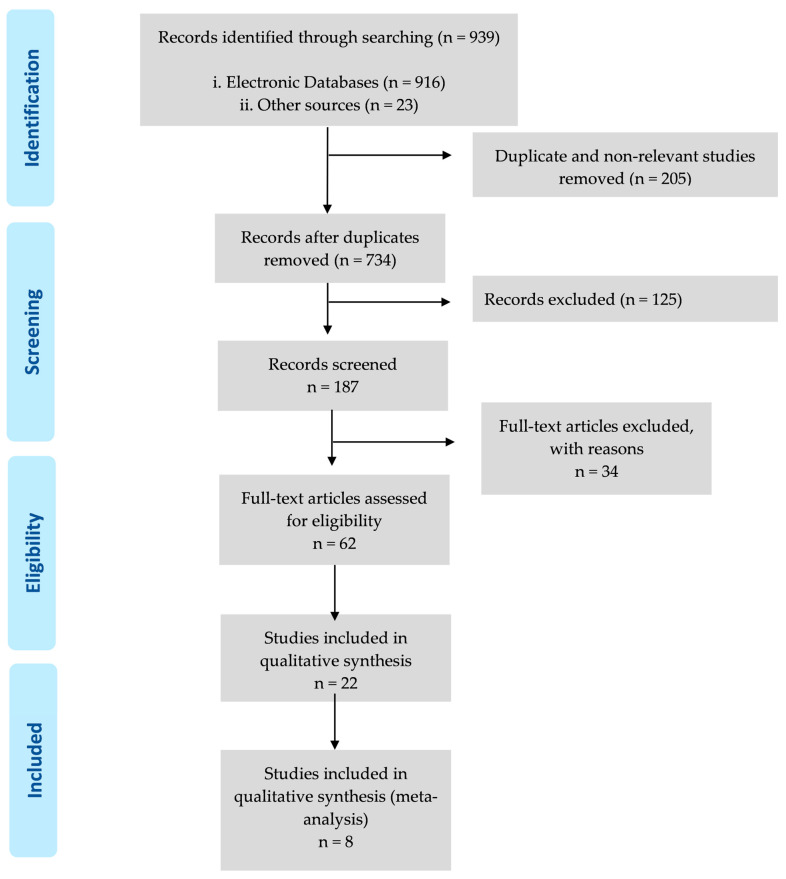
A flow diagram of the work carried out in this study is according to the Systematic Reviews and Meta-Analyses (PRISMA) 2009 recommendations.

As summarized in Figure 1 and Table 1, the search of the electronic databases identified 916 studies, which was reduced to 734 after the 205 studies duplicated across the databases were removed. The search of the grey literature identified 22 studies, which were reduced to 8. Consequently, this search strategy identified 734 unique studies.

The titles of the 734 studies were reviewed to assess their relevance against three exclusion criteria. Where the title provided insufficient information, the abstract was reviewed, or, if required, the complete article was screened. The exclusion criteria were (i) the most recent publications that we could find (287 studies were excluded) and (ii) articles related to humans (124 studies were excluded). In total, 411 studies were excluded based on these criteria. Finally, eight studies were included in this systematic review.

### 2.5. Risk-of-Bias Assessment

The risk of bias was evaluated by comparing each sample to the Critical Assessment Skills Program’s (CASP) Cohort Research Checklist [13]. The study assessed the confounding variables of sample size, age, gender, post-mortem time, and analysis technique within the CASP checklist. The study’s output was rated as “bad”, “fair”, or “good” using the CASP checklist. The evidence was graded as having an excellent, moderate, poor, or extremely low overall quality. According to the CASP risk-of-bias assessment, most studies (75%) were judged as “good” due to the considered variables, while 25% of studies were judged as “poor” or “moderate”, primarily due to confounding variables not being considered. Participants were recruited from a few geographic regions, making it difficult to generalize beyond these regions. Overall, the quality of the literature was “good” (Table 2).

**Table 1:** Characteristics of studies included in the systematic review.

		No. of Analyzed Patients							
GO Arm	Disease	GO Arm	No-GO Arm	Median Age (Range)	Gender (Male %)	Treatment Stage	Chemotherapy	GO Dose and Schedule	Median Follow-Up
Walter et al. 2003 [5]	AML	23	0	-	-	MRP MK-571		No cytotoxicity improvement	
Walter, 2013 [14]	AML	191	0	-	-	Mitoxantrone, etoposide, and cytarabine with or without lintuzumab.	Yes	12 mg/m^2^	ORR with incomplete recuperation of counts of platelets (RCp): 36% vs. 28%, *p* = 0.28
Borthakur et al. 2014 [15]	AML	45		19–76	60	FLAG-GO	Yes	3 mg/m^2^ on day 1.	91% CR and 4% RCp.
Gamis et al. 2014 [16]	AML	511	511	0–29	-	No	Yes	3 mg/m^2^ on day 6 of induction cycle 1	4.1 years (patients survival)
Amadori et al. 2016 [17]	AML	118	119 (BSC)	61–80	-		No	6 mg/m^2^ on day 1 and 3 mg/m^2^ on day 8	4.9 months for GO group and 3.6 months for BSC group.
Olombel et al. 2016 [18]	AML	104	96	62.1 (50–70)	99 (49.5%)			The beneficial effect of GO aggregation was only observed in those patients with high CD331 content.	
Hosono et al. 2021 [19]	AML	19	NO	36–86				9 mg/m^2^ on days 1 and 15	31.6% of patients improved
Döhner et al. 2023 [20]	NPM1-mutated AML	292	-	18–60		Idarubicin, cytarabine, etoposide, and cytarabine.		-	The cumulative incidence of relapse was significantly reduced by gGO (2-year cumulative incidence of relapse, 37% (95% CI: 31–43) in the standard group and 25% (20–30) in the GO group.

No.: number; GO: gemtuzumab ozogamicin; AML: acute myeloid leukemia; ORR: overall response rate; CR: complete remission; RCp: recuperation of counts of platelets.

## 3. Gemtuzumab Ozogamicin

### 3.1. History

Regular marketing approval in the U.S. requires evidence of clinical benefit and an overall improvement in survival or a reduction in disease-related symptoms, provided with adequate, controlled clinical trials. However, accelerated approval is allowed to market drugs to patients with serious illnesses or at risk of death when an improvement in the surrogate endpoint is likely to predict clinical benefit. Thus, these approvals require that additional clinical trials be completed after approval to diligently verify and describe the clinical benefits [7].

GO received accelerated approval in 2000 as a single agent at a dose of 9 mg/m^2^ per day (days 1 and 14) to treat patients with CD33 + AML in their first relapse who were 60 years of age or older and who were not considered for cytotoxic chemotherapy. Wyeth Pharmaceuticals committed to conducting a study to confirm the clinical benefit. However, it was observed that neither response nor survival increased. Interestingly, there was an increase in deaths when adding GO, and Wyeth Pharmaceuticals withdrew GO from the market in 2010 [7]. After this, several clinical trials were carried out that showed some beneficial results, but not enough to say that GO was positive. Until 2013, GO still had no trial confirming its therapeutic effect. Since it was withdrawn, the FDA cautiously allowed access to GO until a full review of the data obtained was completed [7].

It was returned to the market after the FDA’s approval in September 2017 with a new recommended dose, referred to in this article, a different dosing schedule, and a new target population to which it is directed. It was approved after a comprehensive review that showed that the benefits outweighed the risk of toxicity [6].

### 3.2. Gemtuzumab Ozogamicin

GO is an anti-CD33 IgG4 monoclonal antibody, conjugated to dimethylhydrazide N-acetyl-γ1-calicheamicin (calicheamicin DMH), a hydrazide derivative of calicheamicin [9].

Calicheamicin is a natural, hydrophobic enediin antibiotic isolated from the actinomycete Micromonospora echinospora calichensis. Natural enediin antibiotics are a class of unique reactive compounds that, once aromatized, produce cytotoxic biradicals, resulting in the cleavage of phosphodiester bonds in DNA [9]. The conjugation between the antibody and calicheamicin is achieved with a bifunctional linker that allows a better balance of stability in physiological buffers (pH 7.4) and drug release efficiency at lysosomal pH. The calicheamicin/antibody ratio is 2:3, with 50% of the antibody unconjugated [9].

### 3.3. CD33

CD33 is a 67 kDa transmembrane glycoprotein specific to the myeloid lineage, encoded by chromosome 19q13.3. It can be expressed as a homodimer in its physiological state and belongs to the siglec immunoglobulin superfamily, with endogenous receptors that bind sialic acids (Table 3) [14]. It is expressed in the hematopoietic lineage but only manifests in Kupffer cells in the liver and microglia outside the hematological system [21]. Siglecs are involved in cell–cell interactions and hematopoietic and immune system signaling. CD33 contains two immunoglobulin domains, a transmembrane region, and a cytoplasmic tail containing two ITIM-like (inhibitory) sequences [9]. Two tyrosine residues appear in the protein in the cytoplasmic tail. After every three amino acids, there are hydrophobic residues that resemble the ITIM motifs that indicate the structure of many inhibitory receptors. These residues are found at positions 340 and 358 [14].

The phosphorylation of these tyrosine residues allows the recruitment and activation of the tyrosine phosphatases SHP1 and/or SHP2. While SHP1 and SHP2 are recruited to the ITIM340 motif, the ITIM358 motif functions to recruit only SHP2. These tyrosine phosphatases may dephosphorylate CD33 as a possible negative feedback control of CD33 signaling or dephosphorylate and downregulate nearby receptors [14].

SH2 domain-containing suppressor of cytokine signaling 3 (SOCS3) can compete with SHP-1/2 for binding to phosphorylated CD33, leading to ubiquitin recruitment and concomitant accelerated proteasomal degradation of CD33 and SOCS3 [14]. Since CD33 can bind these phosphatases and become phosphorylated, CD33 likely serves as an inhibitory receptor in the myeloid compartment, inhibiting signals produced by ITAM patterns in other receptor systems [21].

In vitro experiments show that CD33 can act as an inhibitory receptor. Furthermore, the addition of an anti-CD33 antibody can induce apoptosis in AML cell cultures. Other studies have documented the internalization of the CD33/anti-CD33 complex by the target cell. These properties allow the use of antibodies directed against CD33 as a treatment for AML, in addition to the fact that CD33 is expressed in approximately 90% of AML cases, as observed with the presence of the antigen in more than 20% of blasts of Leukemic patients [9]. However, for GO to be effective, CD33 expression is required in more than 70% of blasts [18].

Recent studies have emphasized the relationship between genetic variants of CD33 and the effectiveness of GO in patients with acute myeloid leukemia. It has been reported that cutting and splicing variants of the CD33 gene generate alternative isoforms of the transmembrane receptor that compromise the binding of GO. Five additional single-nucleotide polymorphisms (SNPs) in the CD33 gene (rs1803254, rs35112940, rs2455069, rs61736475, and rs201074739) have been identified in patients with AML, which may modulate the anti-leukemic effect of GO [22,23].

### 3.4. Gemtuzumab Ozogamicin Action Mechanism

**Figure 2:**
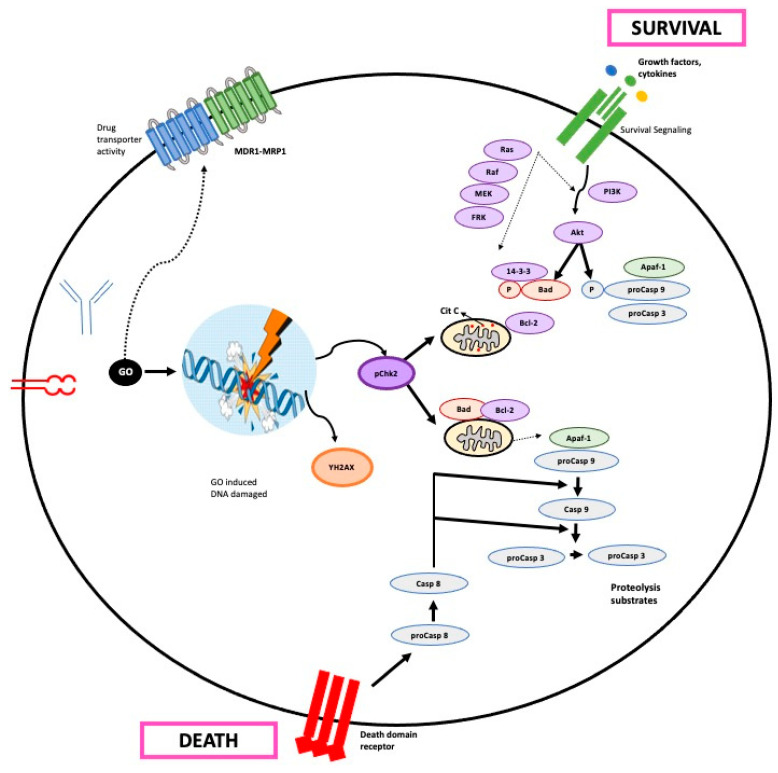
GO action mechanism in the cell. Apaf-1: Apoptosis protease-activating factor-1; bad: Bcl-2 agonist of cell death; FRK: Fyn-related kinase; GO: gemtuzumab ozogamicin; MDR1-MRP1: multidrug resistance-related protein transporter; MEK: Mitogen-activated protein kinase; pChk2: Phospho Checkpoint kinase 2; PI3K: Phosphoinositide 3-kinase; YH2AX: histone gamma H2AX.

GO is targeted rapidly and explicitly to CD33+ cells, forming the GO-CD33 immune complex, and internalized by the cells in an endosome. Thus, GO entry is a function of the number of CD33 molecules on the cell surface (Figure 2) [9]. This internalization mechanism would be CD33-specific, but another CD33-independent mechanism can also occur in malignant cells with endocytic capacity [24].

Following endocytosis, the immune complex unites with a lysosome to liberate the calicheamicin derivative from the antibody and generate a reactive intermediate through reduction with glutathione [9]. This release occurs thanks to the fact that GO incorporates a bifunctional acid-hydrolyzable linker that is stable at physiological pH but is efficiently degraded in the acidic environment of the lysosome [5].

When released, calicheamicin is reduced to a highly reactive 1,4-dehydrobenzene di-radical. At this time, the di-radical can enter the nucleus or be expelled to the outside by a resistance mechanism mediated by the ABC transporter family [14]. If it enters the nucleus, it positions itself in the minor groove of DNA and abstracts hydrogen atoms from the phosphodiester backbone. The resulting radicals seek oxygen and begin a sequence of events that end in DNA’s single- and double-strand cleavages [14].

This DNA damage causes a strong response with a cell cycle arrest in the G2/M phase followed by DNA repair, or, if the damage is very severe, apoptosis and cell death via the mitochondrial route [24]. GO treatment induces the proapoptotic activation of Bak and Bax such that the activation of caspase 3 will lead to cell apoptosis [4].

Calicheamicin-induced double-strand breaks initiate DNA repair by activating the repair protein ATM/ATR and DNA-dependent protein kinase (DNA-PK). In turn, ATM carries out cell cycle arrest in the G2/M phase, activating cyclin B1 and phosphorylating the kinases Chk1 and Chk2 [14]. DNA-PK phosphorylates H2AX in rapid response to double-strand breaks, a step required to recruit DNA damage repair proteins [14].

Multidrug resistance (MDR), mediated by ATP-dependent drug transporters such as P-glycoprotein (PGP) and proteins related to multidrug resistance, is also linked to preclinical responses to GO therapy. Studies in patients treated solely with GO report an association between PGP function (and multidrug resistance protein 1, MrP1), the persistence of blasts in the bone marrow, the inability to achieve complete remission, and/or the reduction in GO-induced in vitro apoptosis (Table 4) [25].

### 3.5. Gemtuzumab Ozogamicin Pharmacokinetics

For GO administration at 9 mg/m^2^ (two doses, 14 days apart), the maximum concentration after the first dose for patients receiving 9 mg/m^2^ GO was 3.0 mg/mL and increased to 3.6 mg/mL after the second dose (Table 5) [26]. Wire et al. [26] performed a review and proposed a table of recommended doses of GO in patients with AML.

N-acetyl gamma calicheamicin dimethylhydrazide is approximately 97% bound to human plasma proteins in vitro. Population pharmacokinetic analyses found that the total volume of antibody distribution was approximately 21.4 L in patients [26].

The antibody clearance value in plasma was 0.35 L/h after the first dose and 0.15 L/h after the second dose, a decrease of approximately 60%. The terminal plasma half-life was 62 h after the first dose and 90 h after the second dose [26]. In vitro studies demonstrated that the calicheamicin derivative is extensively metabolized, primarily through non-enzymatic reduction of the disulfide moiety [26].

Regarding contraindications, its use in pregnant women is not recommended since, after experimental studies in animals, it has been observed that the damage to the fetus was more significant than or equal to the exposure of a dose 0.4 times higher than the usual one. Therefore, its use is not indicated during breastfeeding [26]. Furthermore, other studies in rats have shown that it affects the fertility of both females and males [26].

A study showed that when GO was administered as a single agent, it appeared to be an effective option for treating relapsed AML, with a 31.6% response rate [19]. The same conclusion was reached by another study, which showed that GO had an antileukemic effect in AML patients with NPM1 mutation and significantly reduced the cumulative incidence of recurrence rate. This finding suggests that adding GO could lessen the requirement for rescue medication in these participants [20]. The same conclusion was found by Borthakur et al. [15], whose analyses showed that patients with core binding factor AML who received GO at induction had an overall survival benefit.

Another study analyzed the behavior of GO in children, and although the post-administration analyses did not improve, the risk of relapse was significantly reduced [16]. Amadori et al. observed that the overall survival benefit with GO was especially evident in patients with high CD33 expression status [17].

### 3.6. Gemtuzumab Ozogamicin Resistance Mechanism

GO resistance mechanisms encompass a multifaceted interplay of cellular processes that challenge its therapeutic efficacy.

**Figure 3:**
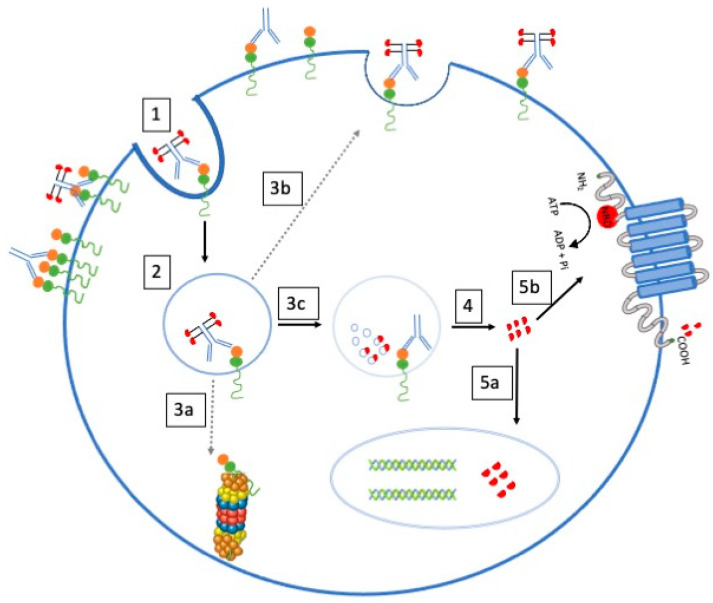
Proteins expressed in the cell can pump antileukemic agents into the extracellular medium. 1—Binding of GO to CD33 on the cell surface. 2—Internalization of CD33/GO Complex into endosome. 3a—EC5/5OCS3-mediated degradation of CD33 via 26S proteasome. 3b—Recycling of GO/CD33 complex to cell surface. 3c—Hydrolytic release of calicheamicin in lysosome. 4—Diffusion of calicheamicin into cytoplasm. 5a—Translocation of calicheamicin into nucleus. 5b—Extrusion of calicheamicin via ABC transporters.

A prominent resistance factor involves multidrug resistance (MDR), where malignant cells develop resistance to various cytotoxic drugs by actively expelling them through membrane transport proteins. The ATP-binding cassette (ABC) superfamily, particularly P-glycoprotein (P-gp), plays a pivotal role in this pharmacological flux (Figure 3). Notably, P-gp, expressed in the blast cells of many AML patients, actively pumps out cytotoxic agents, diminishing intracellular drug accumulation and contributing to GO resistance. Multidrug resistance (MDR) is when malignant cells become resistant to unrelated cytotoxic drugs by expulsing membrane transport protein to the cell exterior [4]. The ATP-binding cassette (ABC) superfamily members, including MDR and MRP subfamily proteins, mediate this pharmacological flux. The best characterized and most intensively studied transporter is P-glycoprotein (P-gp), belonging to the MDR subfamily. [5] P-gp is a membrane glycoprotein that actively pumps cytotoxic agents out of cells and decreases intracellular drug accumulation. It is expressed in many healthy tissues but is also found in the blast cells of 19% to 75% of AML patients [5].

What happens with P-gp is that since it has substrates of similar size to calicheamicin, the latter can have a modulating effect on cytotoxicity. P-gp expression is related to treatment failure in patients receiving GO. Cell lines that overexpress P-gp are resistant to GO, and inhibitors of P-gp function can restore sensitivity to the drug [27]. Furthermore, AML blasts from responders to GO treatment had a higher mean level of CD33 and less P-glycoprotein activity than non-responders, who had an inverted relationship [9].

Cells in the G0 phase of the cell cycle are resistant to GO, whereas those in the G1, S, or G2/M phases appear more susceptible to it. Cells in the G0 phase that are at rest are less vulnerable to the cytotoxic effects of catecholamines [22]. Another resistance mechanism that has been proposed has to do with the saturation state of CD33. Due to the consumption of GO in the peripheral blood and poor ability to enter the bone marrow, high levels of CD33 tumor burden in the peripheral blood and high levels of circulating CD33 confer resistance to the medication and are linked with weaker responses [22]. Other alternative resistance mechanisms include altered pharmacokinetics, reduced ability of GO to bind to leukemic cells, anti-apoptotic mechanisms independent of drug efflux, and anti-apoptotic Bcl-2 proteins [22].

It seems that the main mechanism by which GO causes cell death is through the mitochondrial pathway of apoptosis; resistance to this mechanism is linked to the overexpression of anti-apoptotic proteins such as Bcl-2 and Bcl-XL, which prevent GO from causing damage to cells. Additionally, GO resistance in AML cells has been connected to the in vitro activation of survival signaling pathways such as PI3K/AKT, MEK/ERK, and JAK/STAT [4]. Another contributing factor involves the multidrug resistance protein (MRP1) extruding conjugated and unconjugated organic anions and modulating chemotherapeutic agents’ toxicity in healthy tissues [5]. MRP1 is overexpressed in 7–30% of AML cases; it can also attenuate the cytotoxicity of GO in cell lines and some primary AML cell samples [27].

However, its impact on GO susceptibility seems minor, particularly in the presence of functional P-gp. These intricate resistance mechanisms underscore the need for a comprehensive understanding to devise strategies that enhance GO’s effectiveness in overcoming AML.

Table 6 shows the main findings on GO resistance mechanisms.

### 3.7. Gemtuzumab Ozogamicin Toxicity

GO exhibits a distinct toxicity profile that necessitates careful consideration in clinical applications (Table 7) [15,28]. While it generally presents with lower extramedullary toxicity compared to traditional treatments for AML relapses, GO-induced toxicities are not negligible [28]. Although GO has considerably less extramedullary toxicity than typical treatments for AML relapses, such as high-dose cytarabine, one notable adverse effect is the risk of hepatic veno-occlusive disease (HVOD) and sinusoidal obstructive syndrome, particularly in patients with a high tumor burden [29]. In patients with high tumor burden, the incidence of HVOD after GO therapy at a dose of 9 mg/m^2^ is reported to be around 5–10%. However, GO at lower doses combined with chemotherapy is less toxic [26].

Antibody measurements best represent the pharmacokinetics of GO. After administration, the drug is distributed to a limited space. Slow elimination occurs, followed by a half-life of about 67 h. Apparently, due to a drop in leukemia cells, there is a considerable increase in concentration after the second dose compared to the first. Calicheamicin metabolites can be detected transiently in patients’ serum [9].

The high incidence of hepatotoxicity with GO therapy may reflect the metabolism of the free drug and the damage induced by calicheamicin in the endothelial cell’s sinuosities of the liver once it has been separated from the anti-CD33 antibody. Additionally, infiltration of leukemic blasts may occur in the liver or, less likely, there may be damage to Kupffer cells and sinusoidal cells that are CD33-positive. However, even CD33-negative human hepatocytes can metabolize GO [24].

The IgG4 component of GO is not cytotoxic in vitro, nor does it induce complement-mediated or antibody-dependent cellular cytotoxicity. These antibodies have been used as vehicles to transport the drug [29]. Fever, chills, hypotension, skin rash, hypertension, hyperglycemia, dyspnea, nausea, emesis, and headache are among the side effects of GO [22].

The two most significant toxicities linked to GO are hepatotoxicity and hematological adverse effects. Hematological side effects, including thrombocytopenia and neutropenia, are regularly observed when GO is taken with chemotherapy, although GO has been linked to decreased toxicity at lower dosages. This highlights the necessity of carefully weighing the risks and benefits of GO before using it in clinical settings [22]. An effect on myelopoiesis may occur because pluripotent stem cells do not express CD33, but the most differentiated multipotent cells are CD33+ and may represent a GO target.

Additionally, other reported adverse effects include fever, chills, hypotension, skin rash, hypertension, hyperglycemia, dyspnea, nausea, emesis, and headache, highlighting the importance of monitoring and managing these potential complications during GO treatment.

## 4. Discussion

This systematic review aimed to comprehensively evaluate GO, focusing on its molecular structure, mode of action, pharmacokinetics, recommended dosage, resistance mechanisms, and associated toxicities to provide valuable information on the potential benefits and risks associated with its clinical effect. It reflects on its approval, withdrawal, and subsequent market reintroductions, highlighting the challenges and controversies surrounding its use.

The history of the approval and subsequent withdrawal of GO, followed by its reintroduction in 2017, raises questions about the initial clinical benefits and the need for further trials. Accelerated approval in 2000, based on surrogate endpoints, led to postmarketing studies after observed problems, underscoring the importance of comprehensive clinical trials for drug evaluation. After analyzing the data from the different studies, we can raise the doubt that GO may have more adverse than beneficial effects, although it depends on the population group in which it is administered. Furthermore, as mentioned above, in pregnant women, there is a possibility that it could be teratogenic [24]. However, very beneficial effects were observed in patients over 60 years of age to whom conventional chemotherapy could not be applied, as well as in patients who had suffered a first relapse, so there is some controversy surrounding its application [3]. The accelerated approval by the FDA and its lack of clinical trials have probably contributed to a more negative view of GO since, if different doses had been tested before its marketing, the side effects would have been fewer when establishing the dose, and there may be a greater predisposition to receive the treatment. Perhaps its use in the healthcare field would have favored the use of this drug thanks to more advanced knowledge on the part of patients and greater experience of its effects on them. As a possible alternative, a less toxic use could be considered that could minimize the probability of suffering from EHVOD by applying it together with other chemotherapeutic drugs, since it has been shown that the incidence of this hepatotoxic effect is significantly reduced [22].

The effectiveness of GO is indisputable, since by presenting an antibody against CD33 and being so specific, it will act against leukemic cells that present said antigen on their membrane surface. Its effectiveness has been proven through various clinical trials. However, in terms of toxicity, a dose of 3 mg/m^2^ is what the FDA has established as the recommended dose after verifying that it produces HVOD and sinusoidal obstructive syndrome, in addition to hematological side effects. Therefore, it can be concluded that, although it is an effective treatment, it has some important side effects for the patient, which can aggravate their health. Regarding resistance, given that MDR-Pgp and MRP1 intervene in the expulsion of the drug to the extracellular environment, therefore generating resistance towards GO, the use of inhibitory agents, such as cyclosporine A and the ligand for the peripheral benzodiazepine receptor PK11195, can reverse resistance [9].

To further increase the efficacy and cytotoxicity of GO, AML cells can be exposed to colony-stimulating factor (G-CSF). Furthermore, the histone deacetylase inhibitor valproic acid (VPA) also sensitizes AML cells to GO, reducing the possibility of resistance. The effects of VPA treatment are related to the intercalation of calicheamicin into DNA and increased DNA degradation [9]. A better understanding of the role of glutathione and other redox mediators in calicheamicin DMH activation and/or non-nuclear cell damage mechanisms could provide insights into the enhanced cytotoxicity of GO, including the effective use of the combination of GO with conventional chemotherapeutics agents that decrease intracellular glutathione [25]. Furthermore, because a high amount of CD33 in the blood prevents the entry of GO to the bone marrow, this could be used as an argument for a more personalized dosage [25]. In any case, future studies should focus on the most effective and least toxic dose and schedule of GO and its fractionation.

Although GO has shown promising results in some patients, its application is restricted by various limitations and challenges. Firstly, resistance to GO is a major concern, and it has been observed that specific proteins expressed during the application of the agent can trigger resistance phenomena. A detailed understanding of the molecular mechanisms underlying this resistance is essential to optimize treatment efficacy. Furthermore, the specific expression of CD33 on cancer cells is crucial for the success of GO, as its effectiveness has been shown to depend on high CD33 expression. This requirement limits its application to only those patients whose cancer cells meet this criterion, excluding those with low levels of CD33 expression. Liver toxicity, specifically hepatic veno-occlusive disease (HVOD), has been a significant concern associated with GO. Although dose reduction has mitigated this risk, the incidence of liver toxicity remains a limiting factor in drug administration.

Despite these limitations, GO is beneficial in specific subgroups of patients, such as those over 60 years of age and those who have experienced a first relapse. Identifying and understanding these specific subgroups may be vital to optimizing GO use and improving treatment outcomes. In the future, it is essential to conduct further research to address the identified limitations. This includes extensive studies on resistance mechanisms, dose optimization, and identifying biomarkers that can predict responses to GO. Furthermore, exploring treatment combinations, especially with other chemotherapeutic agents, could be a valuable strategy to improve GO’s efficacy and reduce its associated toxicity. Ultimately, a personalized approach based on understanding the molecular biology of the disease and individual responses to treatment could pave the way for significant advances in treating AML with GO.

Although GO was withdrawn from the market due to its toxicities, different studies have shown that GO is effective in treating patients with AML. This sequence of events highlights the need for adequate study of the dose and schedule of potentially effective medications in AML to minimize the risk of ruling out potentially clinically beneficial medications. Additionally, there are ongoing studies to evaluate the efficacy of GO with chemotherapy or non-chemotherapeutic agents, as well as to eliminate MRD, which may further expand the role of GO in AML [30]. Furthermore, in recent years, advances in sequencing technologies, strategies for monitoring minimal residual disease, genotyping studies, and greater knowledge of the mechanisms of therapeutic resistance have expanded the number and type of biomarkers capable of predicting the effects of GO. Future studies in large cohorts should attempt to combine the various biomarkers to improve prediction accuracy, thus paving the way for precision medicine in real-life clinical practice [31].

## 5. Conclusions

In conclusion, GO stands out as a potential treatment for acute myeloid leukemia (AML), specifically in patients over 60 years of age who are not eligible for conventional chemotherapy or who have experienced a first relapse. The FDA approval history of GO reflects the challenges faced in determining its optimal dosage and use, as it was initially withdrawn from the market due to observed toxicity. However, subsequent studies led to its re-approval in 2017 with a revised dosing schedule, targeting a specific population where the benefits outweigh the risks. The drug’s action mechanism involves its specificity for CD33+ cells, which triggers apoptosis through calicheamicin-induced DNA damage. Despite promising results in some patient groups, resistance mechanisms, including P-glycoprotein expression and antiapoptotic pathways, pose challenges.

Furthermore, the toxicity profile of GO, mainly hepatic veno-occlusive disease, raises concerns. The pharmacokinetics of the drug and recommended doses have been carefully evaluated, emphasizing the need for cautious administration, especially in pregnant women. While GO demonstrates therapeutic efficacy in certain cases of AML, its use requires careful consideration of patient characteristics and potential side effects. Further research and clinical trials are essential to fully refine its application, improve outcomes, and address resistance mechanisms to fully understand its role in leukemia treatment.

## Figures and Tables

**Table 2:** Risk-of-bias assessment.

	Walter et al. 2003 [5]	Walter, 2013 [14]	Borthakur et al. 2014 [15]	Gamis et al. 2014 [16]	Amadori et al. 2016 [17]	Olombel et al. 2016 [18]	Hosono et al. 2021 [19]	Döhner et al. 2023 [20]
Address a clearly focused issue	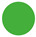	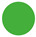	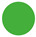	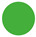	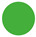	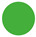	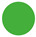	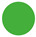
Acceptable cohort recruitment	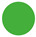	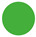	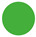	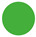	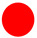	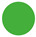	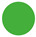	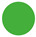
Exposure accurately measured	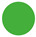	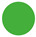	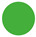	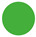	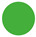	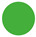	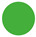	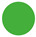
Outcome accurately measured	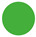	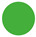	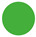	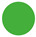	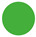	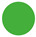	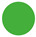	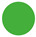
Important confounding factors identified	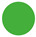	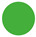	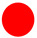	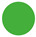	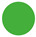	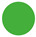	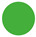	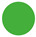
Important confounding factors accounted for	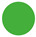	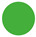	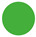	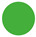	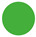	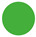	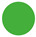	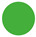
Precise results	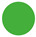	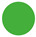	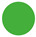	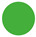	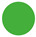	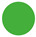	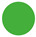	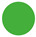
Believable results	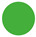	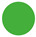	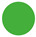	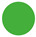	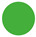	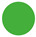	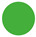	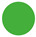
Results fit with other available data.	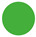	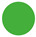	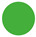	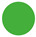	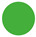	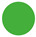	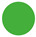	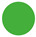
Overall quality score	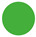	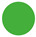	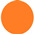	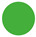	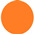	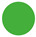	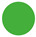	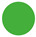

Data based on CASP-based risk-of-bias assessment. Green is a good risk of bias; orange is a moderate risk of bias; red is a low risk of bias.

**Table 3:** Main findings on CD33.

CD33 Overview
CD33 is a 67 kDa transmembrane glycoprotein specific to the myeloid lineage. Encoded by chromosome 19q13.3, it belongs to the siglec immunoglobulin superfamily.
Expression and Structure
Physiological expression as a homodimer. The hematopoietic lineage includes Kupffer cells in the liver and microglia outside the hematopoietic system. Contains two immunoglobulin domains, a transmembrane region, and a cytoplasmic tail with two ITIM-like sequences.
Signal Inhibition Mechanism
Tyrosine residues in the cytoplasmic tail allow phosphorylation and recruitment of tyrosine phosphatases SHP1 and/or SHP2. SHP1/2 dephosphorylate CD33 acts as an inhibitory receptor. SOCS3 competes for binding, leading to ubiquitin recruitment and accelerated proteasomal degradation of CD33 and SOCS3.
Inhibitory Role in Myeloid Compartment
CD33 functions as an inhibitory receptor in the myeloid compartment, likely suppressing signals from other receptor systems.
Anti-CD33 Antibody and AML Treatment
The addition of anti-CD33 antibody induces apoptosis in AML cell cultures. CD33/anti-CD33 complex internalization is observed in target cells.
CD33 Expression and GO Effectiveness
CD33 is expressed in approximately 90% of AML cases. GO requires CD33 expression in more than 70% of blasts for effectiveness.
Genetic Variants and GO Response
Genetic variants and splicing isoforms of CD33 influence the binding of GO. Identified single-nucleotide polymorphisms (SNPs) in the CD33 gene may modulate GO’s anti-leukemic effect.

**Table 4:** Main findings on the GO action mechanism.

GO Targeting Mechanism
GO is targeted rapidly and explicitly to CD33+ cells, forming the GO-CD33 immune complex. Internalization of GO is dependent on the number of CD33 molecules on the cell surface.
Endocytosis and Calicheamicin Release
The GO-CD33 immune complex is endocytosed, leading to fusion with a lysosome. Calicheamicin derivative is released from the antibody in the acidic lysosomal environment. GO incorporates a stable acid-hydrolyzable linker that efficiently degrades the lysosome.
Calicheamicin Activation and Nucleus Entry
Released calicheamicin is reduced to a reactive 1,4-dehydrobenzene di-radical. The di-radical can enter the nucleus, causing DNA damage through hydrogen atom abstraction. DNA damage results in single- and double-strand cleavage
Cellular Response and Apoptosis
Calicheamicin-induced DNA damage causes cell cycle arrest in the G2/M phase. Severe damage leads to apoptosis and cell death through the mitochondrial route. Proapoptotic activation of Bak and Bax, followed by caspase 3 activation, induces cell apoptosis.
DNA Repair Mechanisms
Calicheamicin-induced double-strand breaks initiate DNA repair. Repair protein ATM/ATR and DNA-dependent protein kinase (DNA-PK) are activated. ATM induces cell cycle arrest in G2/M, activating cyclin B1 and phosphorylating kinases Chk1 and Chk2. DNA-PK phosphorylates H2AX, recruiting DNA damage repair proteins.
Multidrug Resistance (MDR)
MDR, mediated by ATP-dependent drug transporters (e.g., P-glycoprotein, PGP), is linked to preclinical responses to GO therapy. Patients with higher PGP function and multidrug resistance protein 1 (MrP1) show resistance to GO, persistence of blasts in bone marrow, and difficulty achieving complete remission. In vitro, MDR is associated with reduced GO-induced apoptosis.

**Table 5:** Recommended dose of GO in patients with AML.

Newly diagnosed CD33-positive AML ^a^ (combination regimen)	1 induction cycle: 3 mg/m^2^ (up to 4.5 mg vial) on days 1, 4, and 7 in combination with daunorubicin and cytarabine.	2 consolidation cycles: 3 mg/m^2^ on day 1 (up to 4.5 mg vial) in combination with daunorubicin and cytarabine
Newly diagnosed CD33-positive AML (single-agent regimen):	1 induction cycle: 6 mg/m^2^ as a single agent on days 1 and 3 mg/m^2^ on day 8.	8 continuation cycles: 2 mg/m^2^ as a single agent on day 1 every 4 weeks.
CD33-positive or relapse-resistant AML (single-agent regimen)	1 single cycle: 3 mg/m^2^ (up to a 4.5 mg vial) on days 1, 4 and 7	

^a^ AML: acute myeloid leukemia.

**Table 6:** Main findings on GO resistance mechanisms.

Multifaceted Resistance Mechanisms
GO resistance involves a complex interplay of cellular processes challenging therapeutic efficacy. Multidrug resistance (MDR) is a prominent factor where malignant cells actively expel cytotoxic drugs through membrane transport proteins, particularly P-glycoprotein (P-gp) of the ATP-binding cassette (ABC) superfamily.
P-glycoprotein (P-gp) and GO Resistance
P-gp actively pumps out cytotoxic agents, reducing intracellular drug accumulation and contributing to GO resistance. P-gp expression is found in blast cells of 19% to 75% of AML patients. Overexpression of P-gp in cell lines leads to GO resistance, while P-gp inhibitors can restore sensitivity. Treatment failure in GO-receiving patients is linked to P-gp expression. Responders to GO treatment have higher CD33 levels and lower P-glycoprotein activity.
Cell Cycle and CD33 Saturation as Resistance Mechanisms
GO toxicity is specific for cells in the G1, S, or G2/M phases, with G0-phase cells being resistant. Resistance is associated with high levels of CD33 tumor load and circulating CD33, limiting GO’s entry to the bone marrow. Alternative resistance mechanisms include altered pharmacokinetics, reduced binding to leukemic cells, anti-apoptotic mechanisms, and Bcl-2 protein involvement.
Mitochondrial Pathway and Signaling Pathways in Resistance
GO-induced cell death primarily involves the mitochondrial pathway of apoptosis. Resistance is associated with the overexpression of anti-apoptotic proteins like Bcl-2 and Bcl-XL. Activation of survival signaling pathways (PI3K/AKT, MEK/ERK, JAK/STAT) are linked to GO resistance in AML cells.
Multidrug Resistance Protein (MRP1) Involvement
MRP1 extrudes organic anions, modulating chemotherapeutic agents’ toxicity in healthy tissues. Overexpressed in 7–30% of AML cases, MRP1 can attenuate GO cytotoxicity in cell lines and some primary AML cell samples.

**Table 7:** Main findings on GO toxicity.

Toxicity Profile
GO has a distinct toxicity profile that requires careful consideration in clinical applications. GO generally exhibits lower extramedullary toxicity compared to traditional treatments for AML relapses.
Hepatic Adverse Effects
Notable adverse effects include the risk of hepatic veno-occlusive disease (HVOD) and sinusoidal obstructive syndrome, particularly in patients with a high tumor burden. The incidence of HVOD after GO therapy at a dose of 9 mg/m^2^ is reported to be around 5–10% in patients with a high tumor burden. Lower doses of GO combined with chemotherapy are associated with reduced toxicity. The high incidence of hepatotoxicity may be linked to the metabolism of the free drug and damage induced by calicheamicin in the liver’s endothelial sinuosities. Hepatotoxicity may result from the infiltration of leukemic blasts in the liver or damage to CD33-positive Kupffer and sinusoidal cells. Even CD33-negative human hepatocytes can metabolize GO.
Pharmacokinetics
Antibody measurements are effective in representing the pharmacokinetics of GO, with a slow elimination process and a half-life of about 67 h.
Cytotoxicity and Vehicle Function
The IgG4 component of GO is not cytotoxic in vitro and does not induce complement-mediated or antibody-dependent cellular cytotoxicity; it serves as a vehicle to transport the drug.
Toxicity Profile
GO has a distinct toxicity profile that requires careful consideration in clinical applications. GO generally exhibits lower extramedullary toxicity compared to traditional treatments for AML relapses.
Adverse effects of GO include fever, chills, hypotension, skin rash, hypertension, hyperglycemia, dyspnea, nausea, emesis, and headache. Hematological side effects and hepatotoxicity are the most significant toxicities associated with GO. Lower doses of GO combined with chemotherapy are linked to reduced hematological side effects, but thrombocytopenia and neutropenia are consistently observed.
Adverse Effects
Adverse effects of GO include fever, chills, hypotension, skin rash, hypertension, hyperglycemia, dyspnea, nausea, emesis, and headache. Hematological side effects and hepatotoxicity are the most significant toxicities associated with GO. Lower doses of GO combined with chemotherapy are linked to reduced hematological side effects, but thrombocytopenia and neutropenia are consistently observed.
Effect on Myelopoiesis
The effect on myelopoiesis may be due to targeting CD33+ in most differentiated multipotent cells, as pluripotent stem cells do not express CD33.
Monitoring and Management
Monitoring and managing potential complications, including adverse effects, are crucial during GO treatment.

## Supplementary Materials

The following supporting information can be downloaded at: https://www.mdpi.com/article/10.3390/biomedicines12010208/s1, Table S1. PRISMA checklist.
